# Discrete Quadratic-Phase Fourier Transform: Theory and Convolution Structures

**DOI:** 10.3390/e24101340

**Published:** 2022-09-23

**Authors:** Hari M. Srivastava, Waseem Z. Lone, Firdous A. Shah, Ahmed I. Zayed

**Affiliations:** 1 Department of Mathematics and Statistics, University of Victoria, Victoria, BC V8W 3R4, Canada; 2 Department of Medical Research, China Medical University Hospital, China Medical University, Taichung 40402, Taiwan; 3 Department of Mathematics and Informatics, Azerbaijan University, 71 Jeyhun Hajibeyli Street, AZ1007 Baku, Azerbaijan; 4 Section of Mathematics, International Telematic University Uninettuno, I-00186 Rome, Italy; 5 Department of Mathematics, University of Kashmir, South Campus, Anantnag 192101, Jammu and Kashmir, India; 6 Department of Mathematical Sciences, DePaul University, Chicago, IL 60614, USA

**Keywords:** quadratic-phase Fourier transform, discerete quadratic-phase Fourier transform, convolution, 46E30, 44A05, 44A35, 94A20

## Abstract

The discrete Fourier transform is considered as one of the most powerful tools in digital signal processing, which enable us to find the spectrum of finite-duration signals. In this article, we introduce the notion of discrete quadratic-phase Fourier transform, which encompasses a wider class of discrete Fourier transforms, including classical discrete Fourier transform, discrete fractional Fourier transform, discrete linear canonical transform, discrete Fresnal transform, and so on. To begin with, we examine the fundamental aspects of the discrete quadratic-phase Fourier transform, including the formulation of Parseval’s and reconstruction formulae. To extend the scope of the present study, we establish weighted and non-weighted convolution and correlation structures associated with the discrete quadratic-phase Fourier transform.

## 1. Introduction

While working on the solution of the heat equation, Saitoh [1] developed an extreme generalization of the classical Fourier transform by invoking the theory of reproducing kernels in the form of quadratic-phase Fourier transform (QPFT). Inspired by the work of Saitoh, Castro et al. [2] studied further possibilities for the quadratic-phase Fourier transform by employing a general quadratic function in the exponent of the novel integral transform. It is worthwhile to mention that QPFT circumscribes several integral transforms, including the classical Fourier, fractional Fourier, Fresnel, linear canonical, and special affine Fourier transforms [3]. As a generalization of the celebrated Fourier transform, the quadratic-phase Fourier transform gained its ground intermittently and profoundly influenced several disciplines of science and engineering, including harmonic analysis, quantum mechanics, differential equations, optics, pattern recognition, and so on [4,5,6,7].

Since most of the practical data are processed at discrete samples, the notion of discrete Fourier transforms (DFTs) has emerged as one of the remarkable concepts in digital signal processing [8]. For instance, in the case of audio video processing, continuous signals are first sampled at discrete time intervals and subsequently the Fourier analysis decomposes the sampled signal into its fundamental periodic constituents of complex exponentials. In recent years, significant progress has been made in the development of discrete Fourier transforms, including the formulation of discrete versions of both the fractional Fourier and linear canonical transforms [9,10]. The aforesaid developments together with the fact that the theory of quadratic-phase Fourier transforms is in its infancy provide an impetus towards the formulation of a discrete analogue of the QPFT. Taking this opportunity, our main goal is to introduce the notion of discrete QPFT and study its fundamental properties including Parseval’s and the inversion formulae.

The notion of convolution is one of the most widely acknoweledged and applied concepts in mathematical and physical sciences [11,12]. The product theorem corresponding to a given convolution operation can be viewed as a manifestation of the behavior of the convolution in the transformed domain. The convolution and correlation theorems in the QPFT domain have been paid considerable attention since its birth [13,14]. However, no discrete version of the convolution or correlation theorems eists in the literture. Taking this opportunity, we formulate convolution and correlation structures associated with the discrete quadratic-phase Fourier transform.

The highlights of the article are pointed out below:

- To introduce a discrete version of the quadratic-phase Fourier transform.
- To study all the mathematical properties of the discrete QPFT.
- To establish a weighted convolution and the corresponding product theorems for the discrete QPFT.
- To formulate a chirp-free convolution and correlation structures associated with the discrete QPFT.

The main content of the paper is organised as follows: In Section 2, we formally recall the fundamentals of quadratic-phase Fourier transform. In Section 3, we introduce the notion of discrete quadratic-phase Fourier transform. Section 4 is devoted to the formulation of the discrete convolution and correlation structures in the context of the quadratic-phase Fourier domains. The article ends with an epilogue in Section 5.

## 2. Quadratic-Phase Fourier Transform

The quadratic-phase Fourier transform (QPFT) is a five-parameter class of integral transform, which encompasses several well-known unitary transformations as well as signal processing and optics-related mathematical operations [3]. Due to the extra degrees of freedom, the QPFT is more flexible than other transforms and is, as such, suitable as well as a powerful tool for investigating deep problems in science and engineering. Here, we present the formal definition of the quadratic-phase Fourier transform followed by the corresponding Parseval and inversion formulae. We have the following definition of theinition quadratic-phase Fourier transform:

**Definition** **1.** *The quadratic-phase Fourier transform of any function $f\in L^{2}\left(R\right)$ with respect to a parametric set $\Lambda =\left(A,B,C,D,E\right),\;B\neq 0$, is denoted as $L_{\Lambda }\left[f\right]\left(\omega \right)$ and is defined by*

(1) $$\begin{array}{c} L_{\Lambda }\left[f\right]\left(\omega \right)=\frac{1}{\sqrt{2\pi }}\int_{R}f\left(t\right)\;Q_{\Lambda }\left(t,\omega \right)\;\mathrm{dt}, \end{array}$$

*where Q(t,ω) denotes the kernel of the quadratic-phase Fourier transform and is given by*

(2) $$\begin{array}{c} Q_{\Lambda }\left(t,\omega \right)=\exp\left\{-i\left(At^{2}+Bt\omega +C\omega^{2}+Dt+E\omega \right)\right\},\;\omega \in R. \end{array}$$

Definition 1 allows us to make the following comments regarding the notion of quadratic-phase Fourier transform:

(i). Choosing the parametric set Λ=(0,1,0,0,0), the QPFT (1) boils down to the classical Fourier transform.
(ii). For $\Lambda =\left(-\cot\theta /2,\csc\theta ,-\cot\theta /2,0,0\right),\;\theta \neq n\pi ,n\in Z$. Then, multiplying (1) with $\sqrt{1-i\cot\theta }$ yields the fractional Fourier transform.
(iii). For the case $\Lambda =\left(-A/2B,1/B,-C/2B,0,0\right)$ and then multiplying (1) with $1/\sqrt{iB}$, Definition 1 turns out to be the linear canonical transform.
(iv). For the collection of parameters $\Lambda =\left(-A/2B,1/B,-D/2B,-p/B,(Dp-Bq)/B\right)$ and multiplying (1) with $e^{iDp^{2}/2B}/\sqrt{iB}$ yields the special affine Fourier transform.

For any $f,g\in L^{2}\left(R\right)$, α,β∈C$k,\omega_{0}\in R$ and λ∈R\{0}, the quadratic-phase Fourier transform defined in (1) satisfies the following properties:

(i). Linearity: $L_{\Lambda }\left[\alpha f+\beta g\right]\left(\omega \right)=\alpha \;L_{\Lambda }\left[f\right]\left(\omega \right)+\beta \;L_{\Lambda }\left[g\right]\left(\omega \right)$,
(ii). Translation:
  $$\begin{array}{cc} L_{\Lambda }\left[f(t-k)\right]\left(\omega \right) & =\exp\left\{i\left(\left(4A^{2}B^{-2}C-A\right)k^{2}+\left(4AB^{-1}C-B\right)\omega k+\left(2AB^{-1}E-D\right)k\right)\right\}\\ \; & \;\times L_{\Lambda }\left[f\right]\left(\omega +2AB^{-1}k\right), \end{array}$$
(iii). Modulation:
  $$\begin{array}{c} L_{\Lambda }\left[e^{i\omega_{0}t}f\left(t\right)\right]\left(\omega \right)=\exp\left\{i\left(C\left(B^{-2}\omega_{0}^{2}-2B^{-1}\omega \omega_{0}\right)\right)-EB^{-1}\omega_{0}\right\}L_{\Lambda }\left[f\right]\left(\omega -B^{-1}\omega_{0}\right), \end{array}$$
(iv). Scaling: $L_{\Lambda }\left[f\left(\frac{t}{\lambda }\right)\right]\left(\omega \right)=\left|\lambda \right|\;F_{\Lambda^{\prime }}\left[f\right]\left(\omega \right),\;\Lambda^{\prime }=\left(\lambda^{2}A,B,\lambda^{-2}C,\lambda D,\lambda^{-1}E\right),$
(v). Parity: $L_{\Lambda }\left[f(-t)\right]\left(\omega \right)=F_{\Lambda^{\prime \prime }}\left[f\right]\left(\omega \right),\;\Lambda^{\prime \prime }=\left(A,B,C,-D,-E\right)$,
(vi). Conjugation: $L_{\Lambda }\left[\bar{f}\right]\left(\omega \right)=\bar{F_{-\Lambda }\left[f\right]\left(\omega \right)},\;-\Lambda =\left(-A,-B,-C,-D,-E\right)$.

The QPFT as defined by (1) is reversible in the sense that the function can be retracted from the transformed space via the inversion formula given by

(3) $$\begin{array}{c} f\left(t\right)=F_{\Lambda }^{-1}\left(L_{\Lambda }\left[f\right]\left(\omega \right)\right)\left(t\right)=\frac{\left|B\right|}{\sqrt{2\pi }}\int_{R}L_{\Lambda }\left[f\right]\left(\omega \right)\;\bar{Q_{\Lambda }\left(t,\omega \right)}\;d\omega . \end{array}$$

Moreover, the Plancheral theorem corresponding to QPFT reads:

(4) $$\begin{array}{c} \langle f_{1},f_{2}\rangle =\left|B\right|\;\langle F_{\Lambda }\left[f_{1}\right],F_{\Lambda }\left[f_{2}\right]\rangle ,\;\forall \;f_{1},f_{2}\in L^{2}\left(R\right). \end{array}$$

## 3. Discrete Quadratic-Phase Fourier Transform

In this section, we formally introduce the notion of discrete QPFT and then study the fundamental properties of the proposed transform, including the orthogonality relation, an inversion formula, and the characterization of range. In the sequel, we derive a direct relationship between the discrete Fourier transform and the discrete QPFT.

To numerically approximate a signal *f* in the QPFT domain, a signal is evaluated at *N* periodic points 0,1,⋯,N−1, in the time domain *t* and the QPFT domain $L_{\Lambda }\left[f\right]\left(\omega \right)$. Therefore, we replace t=nΔt and ω=mΔω in the Definition 1, where *n* and *m* are integers and, Δt and Δω are the periodic sampling intervals in the time and QPFT domains, respectively, satisfying ΔωΔt=2π/BN. We obtain a discrete QPFT $L_{\Lambda }\left(x_{N}\right)\left(m\right)$ of x(n)=f(nΔt) analogous to the discrete Fourier transform by replacing the integral with a finite sum:

$$\begin{array}{c} L_{\Lambda }\left(x_{N}\right)\left(m\right)=\frac{1}{\sqrt{N}}\sum_{n=0}^{N-1}x\left(n\right)\;Q_{\Lambda }\left(n,m\right), \end{array}$$

where the kernal $Q_{\Lambda }\left(n,m\right)$ of transformation is given by

$$\begin{array}{c} Q_{\Lambda }\left(n,m\right)=\exp\left\{-i\left(An^{2}\Delta t^{2}+2\pi nm/N+Cm^{2}\Delta \omega^{2}+Dn\Delta t+Em\Delta \omega \right)\right\}. \end{array}$$

If we replace $Z_{n}$ by the cyclic group of $N^{\mathrm{th}}$ root of unity, then the above discussion can be summarized in the matrix notation as:

$$\begin{array}{c} \left(\begin{array}{c} L_{\Lambda }\left(x_{N}\right)\left(0\right)\\ L_{\Lambda }\left(x_{N}\right)\left(1\right)\\ L_{\Lambda }\left(x_{N}\right)\left(2\right)\\ \vdots \\ L_{\Lambda }\left(x_{N}\right)\left(N-1\right) \end{array}\right)=\frac{1}{\sqrt{N}}W_{N}\left(\begin{array}{c} x(0)\\ x(1)\\ x(2)\\ \vdots \\ x(N-1) \end{array}\right), \end{array}$$

where

$$W_{N}=\left(\begin{array}{ccccc} Q_{\Lambda }\left(0,0\right) & Q_{\Lambda }\left(1,0\right) & Q_{\Lambda }\left(2,0\right) & \cdots & Q_{\Lambda }\left(N-1,0\right)\\ Q_{\Lambda }\left(0,1\right) & Q_{\Lambda }\left(1,1\right) & Q_{\Lambda }\left(2,1\right) & \cdots & Q_{\Lambda }\left(N-1,1\right)\\ Q_{\Lambda }\left(0,2\right) & Q_{\Lambda }\left(1,2\right) & Q_{\Lambda }\left(2,2\right) & \cdots & Q_{\Lambda }\left(N-1,2\right)\\ \vdots & \vdots & \vdots & \ddots & \vdots \\ Q_{\Lambda }\left(0,N-1\right) & Q_{\Lambda }\left(1,N-1\right) & Q_{\Lambda }\left(2,N-1\right) & \cdots & Q_{\Lambda }\left(N-1,N-1\right) \end{array}\right).$$

The formal definition of the discrete QPFT is given below:

**Definition** **2.** *Given a parametric set Λ=(A,B,C,D,E),B≠0, the discrete quadratic-phase Fourier transform $L_{\Lambda }\left(x_{N}\right)\left(m\right)$ of a signal $x_{N}\in \ell^{2}\left(Z_{N}\right)$ is defined by*

(5) $$\begin{array}{c} L_{\Lambda }\left(x_{N}\right)\left(m\right)=\frac{1}{\sqrt{N}}\sum_{n=0}^{N-1}x\left(n\right)\exp\left\{-i\left(An^{2}\Delta t^{2}+\frac{2\pi nm}{N}+Cm^{2}\Delta \omega^{2}+Dn\Delta t+Em\Delta \omega \right)\right\}. \end{array}$$

*Observations:*

(i) For $\Lambda =\left(-A/2B,1/B,-D/2B,0,0\right)$, Definition 2 yields the discrete linear canonical transform [8]:
  (6) $$\begin{array}{c} L_{\Lambda }\left(x_{N}\right)\left(m\right)=\frac{1}{\sqrt{N}}\sum_{n=0}^{N-1}x\left(n\right)\exp\left\{i\left(\frac{An^{2}\Delta t^{2}}{2B}-\frac{2\pi nm}{N}+\frac{Dm^{2}\Delta \omega^{2}}{2B}\right)\right\}. \end{array}$$
(ii) For $\Lambda =\left(-\cot\theta /2,\csc\theta ,-\cot\theta /2,0,0\right),\theta \neq n\pi$, Definition 2 boils down to the discrete fractional Fourier transform [9]:
  (7) $$\begin{array}{c} L_{\Lambda }\left(x_{N}\right)\left(m\right)=\frac{1}{\sqrt{N}}\sum_{n=0}^{N-1}x\left(n\right)\exp\left\{i\left(n^{2}\Delta t^{2}+m^{2}\Delta \omega^{2}\right)\frac{\cot\theta }{2}-\frac{i2\pi nm}{N}\right\}. \end{array}$$
(iii) For $\Lambda =\left(0,1,0,0,0\right)$, Definition 2 reduces to the classical discrete Fourier transform as [3]:
  (8) $$\begin{array}{c} L_{\Lambda }\left(x_{N}\right)\left(m\right)=\frac{1}{\sqrt{N}}\sum_{n=0}^{N-1}x\left(n\right)\exp\left\{\frac{-i2\pi nm}{N}\right\}. \end{array}$$
  Next, we show that the proposed discrete QPFT shares an elegant bond with the dicrete Fourier transform. To meet our intension, we proceed as:
  (9) $$\begin{array}{cc} L_{\Lambda }\left(x_{N}\right)\left(m\right) & =\frac{1}{\sqrt{N}}\sum_{n=0}^{N-1}x\left(n\right)\exp\left\{-i\left(An^{2}\Delta t^{2}+\frac{2\pi nm}{N}+Cm^{2}\Delta \omega^{2}+Dn\Delta t+Em\Delta \omega \right)\right\}\\ \; & =\frac{1}{\sqrt{N}}\exp\left\{-i\left(Cm^{2}\Delta \omega^{2}+Em\Delta \omega \right)\right\}\\ \; & \;\;\times \sum_{n=0}^{N-1}\exp\left\{-i\left(An^{2}\Delta t^{2}+Dn\Delta t\right)\right\}\;x\left(n\right)\exp\left\{\frac{-i2\pi nm}{N}\right\}\\ \; & =\frac{1}{\sqrt{N}}\exp\left\{-i\left(Cm^{2}\Delta \omega^{2}+Em\Delta \omega \right)\right\}\sum_{n=0}^{N-1}y\left(n\right)\exp\left\{\frac{-i2\pi nm}{N}\right\}\\ \; & =\exp\left\{-i\left(Cm^{2}\Delta \omega^{2}+Em\Delta \omega \right)\right\}\;F\left(x_{N}\right)\left(m\right), \end{array}$$
  where $F\left(x_{N}\right)\left(m\right)$ denotes the classical discrete Fourier transform of a signal $X\left(n\right)=\exp\left\{-i\left(An^{2}\Delta t^{2}+Dn\Delta t\right)\right\}\;x\left(n\right)$.

From (9), we observe that the computation of the discrete QPFT corresponds to the following steps:

(i). A product by a chirp signal, that is, $x\left(n\right)\to X\left(n\right)=e^{-i\left(An^{2}\Delta t^{2}+Dn\Delta t\right)}\;x\left(n\right)$.

(ii). A classical discrete Fourier transform, that is, $X\left(n\right)\to F\left(x_{N}\right)\left(m\right)$.

(iii). Another product by a chirp signal, i.e., $F\left(x_{N}\right)\left(m\right)\to L_{\Lambda }\left(x_{N}\right)\left(m\right).$

**Figure 1:**
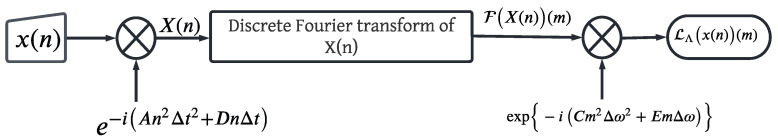
Structure of computing the proposed discrete QPFT.

The aforementioned scheme is depicted in Figure 1.

We now present an example for the lucid illustration of the proposed discrete QPFT given by (5).

**Example** **1.** *Consider a signal x(n)=(1,2,1,3). Then, the discrete QPFT $L_{\Lambda }\left(x_{N}\right)\left(m\right)$ of $x_{n}$ with respect to a parametric set Λ=(A,B,C,D,E) can be computed as:*

*(i)*  *For the parametric set Λ=(0,1,−1,2,1/2), we proceed as:*
$$\begin{array}{cc} \left(\begin{array}{c} L_{\Lambda }\left(x_{N}\right)\left(0\right)\\ L_{\Lambda }\left(x_{N}\right)\left(1\right)\\ L_{\Lambda }\left(x_{N}\right)\left(2\right)\\ L_{\Lambda }\left(x_{N}\right)\left(3\right) \end{array}\right)\; & =\frac{1}{2}\left(\begin{array}{cccc} Q_{\Lambda }\left(0,0\right) & Q_{\Lambda }\left(1,0\right) & Q_{\Lambda }\left(2,0\right) & Q_{\Lambda }\left(3,0\right)\\ Q_{\Lambda }\left(0,1\right) & Q_{\Lambda }\left(1,1\right) & Q_{\Lambda }\left(2,1\right) & Q_{\Lambda }\left(3,1\right)\\ Q_{\Lambda }\left(0,2\right) & Q_{\Lambda }\left(1,2\right) & Q_{\Lambda }\left(2,2\right) & Q_{\Lambda }\left(3,2\right)\\ Q_{\Lambda }\left(0,3\right) & Q_{\Lambda }\left(1,3\right) & Q_{\Lambda }\left(2,3\right) & Q_{\Lambda }\left(3,3\right) \end{array}\right)\left(\begin{array}{c} x(0)\\ x(1)\\ x(2)\\ x(3) \end{array}\right). \end{array}$$
  *Or, equivalently*
$$\begin{array}{c} L_{\Lambda }\left(x_{N}\right)\left(0\right)=\frac{1}{2}\left(1+2\;e^{-2i}+e^{-4i}+3\;e^{-6i}\right)=1.1973-i\;0.1117.\\ L_{\Lambda }\left(x_{N}\right)\left(1\right)=\frac{1}{2}\;\left(0.1109+i\;0.9938\right)\left(1-2\;e^{-2i}-e^{-4i}+3\;e^{-6i}\right)=-1.2417+i\;2.5612.\\ L_{\Lambda }\left(x_{N}\right)\left(2\right)=\frac{1}{2}\;\left(0.9026+i\;0.4303\right)\left(1-2\;e^{-2i}+e^{-4i}-3\;e^{-6i}\right)=-1.1417+i\;0.4178.\\ L_{\Lambda }\left(x_{N}\right)\left(3\right)=\frac{1}{2}\;\left(0.8419-i\;0.5395\right)\left(1+2\;e^{-2i}-e^{-4i}+3\;e^{-6i}\right)=1.0896-i\;1.7297. \end{array}$$
  *That is, the discrete QPFT of x=(1,2,1,3) with respect to a collection Λ=(0,1,−1,2,1/2) is given by $L_{\Lambda }\left(x_{N}\right)=\left(1.1973-i\;0.1117,-1.2417+i\;2.5612,-1.1417+i\;0.4178,1.0896-i\;1.7297\right)$.*
*(ii)*  *For the case Λ=(0,1,0,2,0), we have*
$$\begin{array}{cc} \left(\begin{array}{c} L_{\Lambda }\left(x_{N}\right)\left(0\right)\\ L_{\Lambda }\left(x_{N}\right)\left(1\right)\\ L_{\Lambda }\left(x_{N}\right)\left(2\right)\\ L_{\Lambda }\left(x_{N}\right)\left(3\right) \end{array}\right)\; & =\frac{1}{2}\left(\begin{array}{cccc} Q_{\Lambda }\left(0,0\right) & Q_{\Lambda }\left(1,0\right) & Q_{\Lambda }\left(2,0\right) & Q_{\Lambda }\left(3,0\right)\\ Q_{\Lambda }\left(0,1\right) & Q_{\Lambda }\left(1,1\right) & Q_{\Lambda }\left(2,1\right) & Q_{\Lambda }\left(3,1\right)\\ Q_{\Lambda }\left(0,2\right) & Q_{\Lambda }\left(1,2\right) & Q_{\Lambda }\left(2,2\right) & Q_{\Lambda }\left(3,2\right)\\ Q_{\Lambda }\left(0,3\right) & Q_{\Lambda }\left(1,3\right) & Q_{\Lambda }\left(2,3\right) & Q_{\Lambda }\left(3,3\right) \end{array}\right)\left(\begin{array}{c} x(0)\\ x(1)\\ x(2)\\ x(3) \end{array}\right)\\ \; & =\frac{1}{2}\left(\begin{array}{cccc} 1 & e^{-2i} & e^{-4i} & e^{-6i}\\ 1 & -e^{-2i} & -e^{-4i} & -e^{-6i}\\ 1 & -e^{-2i} & e^{-4i} & -e^{-6i}\\ 1 & e^{-2i} & -e^{-4i} & e^{-6i} \end{array}\right)\left(\begin{array}{c} 1\\ 2\\ 1\\ 3 \end{array}\right). \end{array}$$
  *Or, equivalently*
$$\begin{array}{c} L_{\Lambda }\left(x_{N}\right)\left(0\right)=\frac{1}{2}\left(1+2\;e^{-2i}+e^{-4i}+3\;e^{-6i}\right)=1.1972-i0.1117.\\ L_{\Lambda }\left(x_{N}\right)\left(1\right)=\frac{1}{2}\left(1-2\;e^{-2i}-e^{-4i}-3\;e^{-6i}\right)=-0.1972+i0.1117.\\ L_{\Lambda }\left(x_{N}\right)\left(2\right)=\frac{1}{2}\left(1-2\;e^{-2i}+e^{-4i}-3\;e^{-6i}\right)=-0.8509+i0.8685.\\ L_{\Lambda }\left(x_{N}\right)\left(3\right)=\frac{1}{2}\left(1+2\;e^{-2i}-e^{-4i}+3\;e^{-6i}\right)=1.8509-i0.8685. \end{array}$$
  *That is, the discrete QPFT of x=(1,2,1,3) with respect to a collection Λ=(0,1,−1,2,1/2) is given by $L_{\Lambda }\left(x_{N}\right)=\left(1.1972-i0.1117,-0.1972+i0.1117,-0.8509+i0.8685,1.8509-i0.8685\right)$.*
*(iii)*  *For the collection Λ=(0,1,0,0,0), we obtain*
$$\begin{array}{cc} \left(\begin{array}{c} L_{\Lambda }\left(x_{N}\right)\left(0\right)\\ L_{\Lambda }\left(x_{N}\right)\left(1\right)\\ L_{\Lambda }\left(x_{N}\right)\left(2\right)\\ L_{\Lambda }\left(x_{N}\right)\left(3\right) \end{array}\right)\; & =\frac{1}{2}\left(\begin{array}{cccc} 1 & 1 & 1 & 1\\ 1 & -1 & -1 & 1\\ 1 & -1 & 1 & -1\\ 1 & 1 & -1 & 1 \end{array}\right)\left(\begin{array}{c} 1\\ 2\\ 1\\ 3 \end{array}\right). \end{array}$$
  *Or, equivalently*
$$\begin{array}{c} L_{\Lambda }\left(x_{N}\right)\left(0\right)=\frac{1}{2}\left(1+2+1+3\right)=3.5.\\ L_{\Lambda }\left(x_{N}\right)\left(1\right)=\frac{1}{2}\left(1-2-1+3\right)=0.5.\\ L_{\Lambda }\left(x_{N}\right)\left(2\right)=\frac{1}{2}\left(1-2+1-3\right)=-1.5.\\ L_{\Lambda }\left(x_{N}\right)\left(3\right)=\frac{1}{2}\left(1+2-1+3\right)=2.5. \end{array}$$
  *That is, the discrete QPFT of x=(1,2,1,3) with respect to a collection Λ=(0,1,−1,2,1/2) is given by $L_{\Lambda }\left(x_{N}\right)=\left(3.5,0.5,-1.5,2.5\right)$.*

In the following theorem, we assemble some basic properties of the discrete QPFT (5).

**Theorem** **1.** *For any $x_{N},y_{N}\in \ell^{2}\left(Z_{N}\right)$, α,β,k∈R and λ,μ∈N, the discrete QPFT *(5)* satisfies the following properties:*

(i). *Linearity: $L_{\Lambda }\left(\alpha \;x_{N}+\beta \;y_{N}\right)\left(m\right)=\alpha \;L_{\Lambda }\left(x_{N}\right)\left(m\right)+\beta \;L_{\Lambda }\left(y_{N}\right)\left(m\right)$,*
(ii). *Translation:*
$$L_{\Lambda }\left(x_{N-k}\right)\left(m\right)=\exp\left\{-i\left(Ak^{2}\Delta t^{2}+\frac{2\pi km}{N}+Dk\Delta t\right)\right\}L_{\Lambda }\left(e^{-2iAnk\Delta t^{2}}x_{N}\right)\left(m\right),$$
(iii). *Modulation:*
$$L_{\Lambda }\left(e^{i2\pi \mu n/N}\;x_{N}\right)\left(m\right)=\exp\left\{i\left(C\left(\mu^{2}-2m\mu \right)\Delta \omega^{2}-E\mu \Delta \omega \right)\right\}L_{\Lambda }\left(x_{N}\right)\left(m-\mu \right),$$
(iv). *Scaling:*
$$\begin{array}{c} F_{\Lambda }\left(x_{\lambda N}\right)\left(m\right)=\exp\left\{i\left(C\left(\frac{m^{2}}{\lambda^{2}}-m^{2}\right)\Delta \omega^{2}+E\left(\frac{m}{\lambda }-m\right)\Delta \omega \right)\right\}L_{\Lambda^{\prime }}\left(x_{N}\right)\left(\frac{m}{\lambda }\right), \end{array}$$
  *where $\Lambda^{\prime }=\left(\frac{A}{\lambda^{2}},B,C,\frac{D}{\lambda },E\right)$,*
(v). *Conjugation: $L_{\Lambda }\left(\bar{x_{N}}\right)\left(m\right)=\exp\left\{-2iEm\Delta \omega \right\}\bar{L_{-\Lambda }\left(x_{N}\right)\left(m\right)}$.*

**Proof.**  (i) The proof of linearity property directly follows from the Definition 2.

(ii) To study the effect of discrete QPFT under translation, we proceed as:

$$\begin{array}{cc} \; & L_{\Lambda }\left(x_{N-k}\right)\left(m\right)\\ \; & \;=\frac{1}{\sqrt{N}}\sum_{n=0}^{N-1}x\left(n-k\right)\exp\left\{-i\left(An^{2}\Delta t^{2}+\frac{2\pi nm}{N}+Cm^{2}\Delta \omega^{2}+Dn\Delta t+Em\Delta \omega \right)\right\}\\ \; & \;=\frac{1}{\sqrt{N}}\sum_{u=0}^{N-1}x\left(u\right)\exp\left\{-i\left(A{\left(u+k\right)}^{2}\Delta t^{2}+\frac{2\pi nm}{N}+Cm^{2}\Delta \omega^{2}+D\left(u+k\right)\Delta t+Em\Delta \omega \right)\right\}\\ \; & \;=\frac{1}{\sqrt{N}}\exp\left\{-i\left(Ak^{2}\Delta t^{2}+\frac{2\pi km}{N}+Dk\Delta t\right)\right\}\\ \; & \;\;\times \sum_{u=0}^{N-1}e^{-2iAuk\Delta t^{2}}x\left(u\right)\exp\left\{-i\left(Au^{2}\Delta t^{2}+\frac{2\pi um}{N}+Cm^{2}\Delta \omega^{2}+Du\Delta t+Em\Delta \omega \right)\right\}\\ \; & \;=\exp\left\{-i\left(Ak^{2}\Delta t^{2}+\frac{2\pi km}{N}+Dk\Delta t\right)\right\}L_{\Lambda }\left(e^{-2iAnk\Delta t^{2}}x_{N}\right)\left(m\right). \end{array}$$

(iii) Invoking Definition 2, we have

$$\begin{array}{cc} \; & L_{\Lambda }\left(e^{i2\pi \mu n/N}\;x_{N}\right)\left(m\right)\\ \; & \;=\frac{1}{\sqrt{N}}\sum_{n=0}^{N-1}e^{i2\pi \mu n/N}\;x\left(n\right)\exp\left\{-i\left(An^{2}\Delta t^{2}+\frac{2\pi nm}{N}+Cm^{2}\Delta \omega^{2}+Dn\Delta t+Em\Delta \omega \right)\right\}\\ \; & \;=\frac{1}{\sqrt{N}}\sum_{n=0}^{N-1}x\left(n\right)\exp\left\{-i\left(An^{2}\Delta t^{2}+\frac{2\pi n(m-\mu )}{N}+Cm^{2}\Delta \omega^{2}+Dn\Delta t+Em\Delta \omega \right)\right\}\\ \; & \;=\frac{1}{\sqrt{N}}\exp\left\{i\left(C\left(\mu^{2}-2m\mu \right)\Delta \omega^{2}-E\mu \Delta \omega \right)\right\}\\ \; & \;\times \sum_{n=0}^{N-1}x\left(n\right)\exp\left\{-i\left(An^{2}\Delta t^{2}+\frac{2\pi n(m-\mu )}{N}+C{\left(m-\mu \right)}^{2}\Delta \omega^{2}+Dn\Delta t+E\left(m-\mu \right)\Delta \omega \right)\right\}\\ \; & \;=\exp\left\{i\left(C\left(\mu^{2}-2m\mu \right)\Delta \omega^{2}-E\mu \Delta \omega \right)\right\}\;L_{\Lambda }\left(x_{N}\right)\left(m-\mu \right). \end{array}$$

(iv) Implementing the definition of discrete QPFT (5), we have

$$\begin{array}{cc} \; & F_{\Lambda }\left(x_{\lambda N}\right)\left(m\right)\\ \; & \;=\frac{1}{\sqrt{N}}\sum_{n=0}^{N-1}x\left(\lambda \;n\right)\exp\left\{-i\left(An^{2}\Delta t^{2}+\frac{2\pi nm}{N}+Cm^{2}\Delta \omega^{2}+Dn\Delta t+Em\Delta \omega \right)\right\}\\ \; & \;=\frac{1}{\sqrt{N}}\sum_{u=0}^{N-1}x\left(u\right)\exp\left\{-i\left(\left(A/\lambda^{2}\right)u^{2}\Delta t^{2}+\frac{2\pi n(m/\lambda )}{N}+Cm^{2}\Delta \omega^{2}+\left(D/\lambda \right)u\Delta t+Em\Delta \omega \right)\right\}\\ \; & \;=\frac{1}{\sqrt{N}}\exp\left\{-i\left(Cm^{2}\Delta \omega^{2}+Em\Delta \omega \right)\right\}\exp\left\{i\left(C{\left(m/\lambda \right)}^{2}\Delta \omega^{2}+E\left(m/\lambda \right)\Delta \omega \right)\right\}\\ \; & \;\times \sum_{u=0}^{N-1}x\left(u\right)\exp\left\{-i\left(\left(A/\lambda^{2}\right)u^{2}\Delta t^{2}+\frac{2\pi n(m/\lambda )}{N}+C{\left(m/\lambda \right)}^{2}\Delta \omega^{2}+\left(D/\lambda \right)u\Delta t+E\left(m/\lambda \right)\Delta \omega \right)\right\}\\ \; & \;=\exp\left\{i\left(C\left(\frac{m^{2}}{\lambda^{2}}-m^{2}\right)\Delta \omega^{2}+E\left(\frac{m}{\lambda }-m\right)\Delta \omega \right)\right\}L_{\Lambda^{\prime }}\left(x_{N}\right)\left(\frac{m}{\lambda }\right), \end{array}$$

where $\Lambda^{\prime }=\left(\frac{A}{\lambda^{2}},B,C,\frac{D}{\lambda },E\right)$.

(v) The conjugation property directly follows from the definition (5).

This completes the proof of Theorem 1.  □

In our next theorem, we demonstrate that the discrete QPFT $L_{\Lambda }\left(x_{N}\right)\left(m\right)$ of any signal $x\left(n\right)\in \ell^{2}\left(Z_{n}\right)$ is reversible.

**Theorem** **2.** *If $L_{\Lambda }\left(x_{N}\right)\left(m\right)$ is the discrete QPFT of any arbitrary sequence $x_{N}$, then $x_{N}$ can be reconstructed via:*

(10) $$\begin{array}{c} x_{N}=\frac{1}{\sqrt{N}}\sum_{n=0}^{N-1}L_{\Lambda }\left(x_{N}\right)\left(m\right)\exp\left\{i\left(An^{2}\Delta t^{2}+\frac{2\pi nm}{N}+Cm^{2}\Delta \omega^{2}+Dn\Delta t+Em\Delta \omega \right)\right\}. \end{array}$$

**Proof.**  By virtue of Definition 2, we have

$$\begin{array}{cc} \; & \sum_{v=0}^{N-1}L_{\Lambda }\left(x_{N}\right)\left(m\right)\exp\left\{i\left(Av^{2}\Delta t^{2}+\frac{2\pi vm}{N}+Cm^{2}\Delta \omega^{2}+Dv\Delta t+Em\Delta \omega \right)\right\}\\ \; & \;=\frac{1}{\sqrt{N}}\sum_{v=0}^{N-1}\left(\sum_{n=0}^{N-1}x\left(n\right)\exp\left\{-i\left(An^{2}\Delta t^{2}+\frac{2\pi nm}{N}+Cm^{2}\Delta \omega^{2}+Dn\Delta t+Em\Delta \omega \right)\right\}\right)\\ \; & \;\;\times \exp\left\{i\left(Av^{2}\Delta t^{2}+\frac{2\pi vm}{N}+Cm^{2}\Delta \omega^{2}+Dv\Delta t+Em\Delta \omega \right)\right\}\\ \; & \;=\frac{1}{\sqrt{N}}\sum_{v=0}^{N-1}\sum_{n=0}^{N-1}x\left(n\right)\exp\left\{-i\left(A\left(n^{2}-v^{2}\right)\Delta t^{2}+\frac{2\pi (n-v)m}{N}+D\left(n-v\right)\Delta t\right)\right\}. \end{array}$$

Using the sum

$$\begin{array}{c} \sum_{v=0}^{N-1}\sum_{n=0}^{N-1}\exp\left\{-i\left(A\left(n^{2}-v^{2}\right)\Delta t^{2}+\frac{2\pi (n-v)m}{N}+D\left(n-v\right)\Delta t\right)\right\}=\left\{\begin{array}{cc} N, & n=v\\ 0, & n\neq v, \end{array}\right. \end{array}$$

we obtain

$$\begin{array}{c} \sum_{v=0}^{N-1}L_{\Lambda }\left(x_{N}\right)\left(m\right)\exp\left\{i\left(Av^{2}\Delta t^{2}+\frac{2\pi vm}{N}+Cm^{2}\Delta \omega^{2}+Dv\Delta t+Em\Delta \omega \right)\right\}=\sqrt{N}\;x\left(v\right). \end{array}$$

Or, equivalently

$$\begin{array}{c} x_{N}=\frac{1}{\sqrt{N}}\sum_{n=0}^{N-1}L_{\Lambda }\left(x_{N}\right)\left(m\right)\exp\left\{i\left(An^{2}\Delta t^{2}+\frac{2\pi nm}{N}+Cm^{2}\Delta \omega^{2}+Dn\Delta t+Em\Delta \omega \right)\right\}. \end{array}$$

This completes the proof of Theorem 2.  □

Towards the culmination of this section, we derive the Plancheral formula for the proposed discrete QPFT given by (5).

**Theorem** **3.** *For any finite sequence $x_{N}\in \ell^{2}\left(Z_{N}\right)$, we have*

(11) $$\begin{array}{c} \sum_{m=0}^{M-1}|L_{\Lambda }\left(x_{N}\right)\left(m\right){|}^{2}=\frac{1}{N}\sum_{n=0}^{N-1}|x\left(n\right){|}^{2}. \end{array}$$

**Proof.**  Invoking the definition of discrete QPFT, we have

$$\begin{array}{cc} \; & \sum_{m=0}^{M-1}|L_{\Lambda }\left(x_{N}\right)\left(m\right){|}^{2}\\ \; & \;=\sum_{m=0}^{M-1}L_{\Lambda }\left(x_{N}\right)\left(m\right)\;\bar{L_{\Lambda }\left(x_{N}\right)\left(m\right)}\\ \; & \;=\frac{1}{N}\sum_{m=0}^{M-1}\left(\sum_{n=0}^{N-1}x\left(n\right)\exp\left\{-i\left(An^{2}\Delta t^{2}+\frac{2\pi nm}{N}+Cm^{2}\Delta \omega^{2}+Dn\Delta t+Em\Delta \omega \right)\right\}\right)\\ \; & \;\;\times \bar{\left(\sum_{n=0}^{N-1}x\left(v\right)\exp\left\{-i\left(Av^{2}\Delta t^{2}+\frac{2\pi vm}{N}+Cm^{2}\Delta \omega^{2}+Dv\Delta t+Em\Delta \omega \right)\right\}\right)}\\ \; & \;=\frac{1}{N}\sum_{n=0}^{N-1}\sum_{v=0}^{N-1}x\left(n\right)\;\bar{x(v)}\;\exp\left\{-i\left(A\left(n^{2}-v^{2}\right)\Delta t^{2}+\frac{2\pi (n-v)m}{N}+D\left(n-v\right)\Delta t\right)\right\}. \end{array}$$

Taking v=n, we obtain

$$\begin{array}{c} \sum_{m=0}^{M-1}|L_{\Lambda }\left(x_{N}\right)\left(m\right){|}^{2}=\frac{1}{N}\sum_{n=0}^{N-1}|x\left(n\right){|}^{2}. \end{array}$$

This completes the proof of Theorem 3.  □

## 4. Convolution and Correlation Structures Associated with the Discrete Quadratic-Phase Fourier Transform

The notion of convolution is one of the widely applied concepts in mathematics, with application areas ranging from functional analysis to different fields of signal and image processing, including quantum mechanics, operator theory, pattern recognition, and signal detection [11,12]. Primarily, we formulate a duo of important convolution operations associated with the discrete QPFT (5) and also investigate the fundamental properties.

(i). *Weighted Discrete Convolution in the Quadratic-phase Fourier Domain*

In this subsection, we shall introduce the notion of convolution and correlation structures associated with discrete QPFT which upholds the classical convolution theorem in the sense that, except for a chirp, the discrete QPFT of two convoluted signals corresponds to the product of their respective discrete QPFTs. Nevertheless, we also demonstrate that such a convolution structure satisfies the fundamental properties of commutativity, associativity, and distributivity.

Next, we shall study the convolution operation of two sequences $x_{N},y_{N}\in \ell^{2}\left(Z_{N}\right)$ in the quadratic-phase Fourier domains.

**Definition** **3.** *For a pair of sequences $x_{N}$ and $y_{N}$ belonging to $\ell^{2}\left(Z_{N}\right)$, the discrete quadratic-phase convolution is denoted by ${⊛}_{\Lambda }$ and is defined by*

(12) $$\begin{array}{c} \left(x_{N}{⊛}_{\Lambda }y_{N}\right)\left(u\right)=\sum_{n=0}^{N-1}x\left(n\right)\;y\left(u-n\right)\exp\left\{-i2An\left(n-u\right)\;\Delta t^{2}\right\}. \end{array}$$

In the following, we assemble some properties of the convolution operator ${⊛}_{\Lambda }$ defined by (12).

**Theorem** **4.** *For any sequences $x_{N},y_{N},z_{N}\in \ell^{2}\left(Z_{N}\right)$ and k,α∈N, the discrete quadratic-phase convolution ${⊛}_{\Lambda }$ has the following properties:*

(i). *Commutativity: $\left(x_{N}{⊛}_{\Lambda }y_{N}\right)\left(u\right)=\left(y_{N}{⊛}_{\Lambda }x_{N}\right)\left(u\right),$*
(ii). *Associativity: $\left(x_{N}{⊛}_{\Lambda }y_{N}\right){⊛}_{\Lambda }z_{N}=x_{N}{⊛}_{\Lambda }\left(y_{N}{⊛}_{\Lambda }z_{N}\right),$*
(iii). *Translation: $\left(x_{N}{⊛}_{\Lambda }y_{N}\right)\left(u-k\right)=e^{-i4Ank}\left(e^{i4Anu}u_{N-k}\;{⊛}_{\Lambda }y_{N}\right)\left(u\right),$*
(iv). *Reflection: $\left(x_{N}{⊛}_{\Lambda }y_{N}\right)\left(-u\right)=\left(x_{-N}{⊛}_{\Lambda }y_{-N}\right)\left(u\right),$*
(v). *Scaling: $\left(x_{N}{⊛}_{\Lambda }y_{N}\right)\left(\alpha u\right)=\left|\alpha \right|\left(x_{\alpha N}{⊛}_{\Lambda }y_{\alpha N}\right)\left(u\right).$*

**Proof.**  For the sake of brevity, we omit the proof.  □

In our next theorem, we obtain a convolution theorem associated with the discrete QPFT (5).

**Theorem** **5.** *For any $x_{N},y_{N}\in \ell^{2}\left(Z_{N}\right)$, we have*

(13) $$\begin{array}{c} L_{\Lambda }\left(x_{N}{⊛}_{\Lambda }y_{N}\right)\left(m\right)=\sqrt{N}\exp\left\{i\left(Cm^{2}\Delta \omega^{2}+Em\Delta \omega \right)\right\}\;L_{\Lambda }\left(x_{N}\right)\left(m\right)\;L_{\Lambda }\left(y_{N}\right)\left(m\right). \end{array}$$

**Proof.**  Invoking the definition of discrete QPFT (5), we have

$$\begin{array}{cc} \; & L_{\Lambda }\left(x_{N}{⊛}_{\Lambda }y_{N}\right)\left(m\right)\\ \; & \;=\frac{1}{\sqrt{N}}\sum_{n=0}^{N-1}\left(x_{U}{⊛}_{\Lambda }y_{U}\right)\left(n\right)\exp\left\{-i\left(An^{2}\Delta t^{2}+\frac{2\pi nm}{N}+Cm^{2}\Delta \omega^{2}+Dn\Delta t+Em\Delta \omega \right)\right\}\\ \; & \;=\frac{1}{\sqrt{N}}\sum_{n=0}^{N-1}\sum_{u=0}^{U-1}x\left(u\right)\;y\left(n-u\right)\exp\left\{-i2Au\left(u-n\right)\;\Delta t^{2}\right\}\\ \; & \;\;\times \exp\left\{-i\left(An^{2}\Delta t^{2}+\frac{2\pi nm}{N}+Cm^{2}\Delta \omega^{2}+Dn\Delta t+Em\Delta \omega \right)\right\}\\ \; & \;=\frac{1}{\sqrt{N-U}}\sum_{n=0}^{N-1}\;\sum_{v=0}^{N-U-1}x\left(z\right)\;y\left(v\right)\exp\left\{i2Auv\;\Delta t^{2}\right\}\\ \; & \;\;\times \exp\left\{-i\left(A{\left(u+v\right)}^{2}\Delta t^{2}+\frac{2\pi (u+v)m}{N}+Cm^{2}\Delta \omega^{2}+D\left(u+v\right)\Delta t+Em\Delta \omega \right)\right\}\\ \; & \;=\sum_{n=0}^{N-1}x\left(u\right)\exp\left\{-i\left(Au^{2}\Delta t^{2}+\frac{2\pi um}{N}+Du\Delta t\right)\right\}\\ \; & \;\;\times \frac{1}{\sqrt{N-U}}\sum_{v=0}^{N-U-1}y\left(v\right)\exp\left\{-i\left(Av^{2}\Delta t^{2}+\frac{2\pi vm}{N}+Cm^{2}\Delta \omega^{2}+Dv\Delta t+Em\Delta \omega \right)\right\}\\ \; & \;=\sqrt{N}\exp\left\{i\left(Cm^{2}\Delta \omega^{2}+Em\Delta \omega \right)\right\}\;L_{\Lambda }\left(x_{N}\right)\left(m\right)\;L_{\Lambda }\left(y_{N}\right)\left(m\right). \end{array}$$

This completes the proof of Theorem 5.  □

Next, we introduce the notion of discrete quadratic-phase correlation and then present the corresponding correlation theorem.

**Definition** **4.** *Given a pair of sequneces $x,y\in \ell^{2}\left(Z_{N}\right)$, the discrete quadratic-phase correlation is denoted by ${☆}_{\Lambda }$ and is defined as*

(14) $$\begin{array}{c} \left(x_{N}{☆}_{\Lambda }y_{N}\right)\left(u\right)=\sum_{n=0}^{N-1}x\left(n\right)\;\bar{y(n-u)}\;\exp\left\{i2Au\left(u-n\right)\;\Delta t^{2}\right\}. \end{array}$$

**Theorem** **6.** *For any $x,y\in \ell^{2}\left(Z_{n}\right)$, we have*

(15) $$\begin{array}{c} L_{\Lambda }\left(x_{N}{☆}_{\Lambda }y_{N}\right)\left(m\right)=\sqrt{N}\exp\left\{-i\left(Cm^{2}\Delta \omega^{2}+Em\Delta \omega \right)\right\}\;L_{\Lambda }\left(x_{N}\right)\left(m\right)\;\bar{L_{\Lambda }\left(y_{N}\right)\left(m\right)}. \end{array}$$

**Proof.**  The proof can be obtained in a manner similar to Theorem 5 and is therefore omitted.  □

(ii). *Chirp-free Discrete Convolution in the Quadratic-phase Fourier Domain*

In this subsection, we formulate a notion of chirp-free discrete quadratic-phase convolution operation, which states that the discrete QPFT of convolution of two sequences belonging to $\ell^{2}\left(Z_{n}\right)$ is equal to the product of discrete QPFT and the classical discrete Fourier transform, respectively. The name chirp-free is coined due to the fact that the associated product theorem does not contain any chirp multiplier. In continuation, we shall demonstrate that such a convolution does not satisfy the commutativity and associative properties, however, the distributive property holds good.

**Definition** **5.** *Given a collection of parameters $\Lambda =\left(A,B,C,D,E\right)$ and a pair of sequneces $x_{N},y_{N}\in \ell^{2}\left(Z_{N}\right)$, the chirp-free convolution associated with the discrete QPFT is denoted by ${⊙}_{\Lambda }$ and is defined as*

(16) $$\begin{array}{c} \left(x_{N}{⊙}_{\Lambda }y_{N}\right)\left(u\right)=\sum_{n=0}^{N-1}x\left(n\right)\;y\left(u-n\right)\exp\left\{iA\left(u^{2}-n^{2}\right)\Delta t^{2}\right\}. \end{array}$$

Some important characteristics of the discrete quadratic-phase convolution operation (16) are assembled in the following theorem.

**Theorem** **7.** *For any sequences $x_{N},y_{N},z_{N}\in \ell^{2}\left(Z_{N}\right)$ and the scalars α,k∈N, the discrete quadratic-phase convolution operation ${⊙}_{\Lambda }$ defined in *(16)* satisfies the following properties:*

(i). *Non-commutativity: $\left(x_{N}{⊙}_{\Lambda }y_{N}\right)\left(u\right)\neq \left(y_{N}{⊙}_{\Lambda }x_{N}\right)\left(u\right),$*
(ii). *Non-Associativity: $\left(\left(x_{N}{⊙}_{\Lambda }y_{N}\right){⊙}_{\Lambda }z_{N}\right)\left(u\right)\neq \left(x_{N}{⊙}_{\Lambda }\left(y_{N}{⊙}_{\Lambda }z_{N}\right)\right)\left(u\right),$*
(iii). *Distributivity: $\left(x_{N}{⊙}_{\Lambda }\left(y_{N}+z_{N}\right)\right)\left(u\right)=\left(x_{N}{⊙}_{\Lambda }y_{N}\right)\left(u\right)+\left(x_{N}{⊙}_{\Lambda }z_{N}\right)\left(u\right),$*
(iv). *Translation: $\left(x_{N}{⊙}_{\Lambda }y_{N}\right)\left(u-k\right)=\left(x_{N-k}{⊙}_{\Lambda }y_{N}^{2,\Lambda }\right)\left(u\right),\;y_{N}^{2,\Lambda }\left(t\right)=e^{-2iAkn\Delta t}y\left(n\right),$*
(v). *Scaling: $\left(x_{N}{⊙}_{\Lambda }y_{N}\right)\left(\alpha u\right)=\alpha \left(x_{\alpha \;N}{⊙}_{\Lambda^{\prime }}y_{\alpha \;N}\right)\left(u\right),\;\Lambda^{\prime }=\left(\alpha^{2}A,B,C,\alpha D,E\right),$*
(vi). *Parity: $\left(x_{N}{⊙}_{\Lambda }y_{N}\right)\left(-u\right)=\left(x_{-N}{⊙}_{\Lambda }y_{-N}\right)\left(u\right)$.*

**Proof.**  For the sake of convenience, we omitt the proof.  □

In the following theorem, we demonstrate that indeed the convolution theorem pertaining to the discrete quadratic-phase convolution operation ${⊙}_{\Lambda }$ defined in (16) is chirp-free.

**Theorem** **8.** *For any sequences $x_{N},y_{N}\in \ell^{2}\left(Z_{N}\right)$, we have*

(17) $$\begin{array}{cc} \; & L_{\Lambda }\left(x_{N}{⊙}_{\Lambda }y_{N}\right)\left(m\right)=\sqrt{N}\;L_{\Lambda }\left(x_{N}\right)\left(m\right)\;F\left(e^{-iDn\Delta t}\;y_{N}\right)\left(m\right), \end{array}$$

*where $F\left(x\right)$ represents the classical discrete Fourier transform.*

**Proof.**  Invoking Definition 2, we can compute the discrete QPFT corresponding to (16) as follows:

$$\begin{array}{cc} \; & L_{\Lambda }\left(x_{N}{⊙}_{\Lambda }y_{N}\right)\left(m\right)\\ \; & \;=\frac{1}{\sqrt{N}}\sum_{n=0}^{N-1}\left(x_{U}{⊙}_{\Lambda }y_{U}\right)\left(n\right)\exp\left\{-i\left(An^{2}\Delta t^{2}+\frac{2\pi nm}{N}+Cm^{2}\Delta \omega^{2}+Dn\Delta t+Em\Delta \omega \right)\right\}\\ \; & \;=\frac{1}{\sqrt{N}}\sum_{n=0}^{N-1}\sum_{u=0}^{U-1}x\left(u\right)\;y\left(n-u\right)\exp\left\{iA\left(n^{2}-u^{2}\right)\;\Delta t^{2}\right\}\\ \; & \;\;\times \exp\left\{-i\left(An^{2}\Delta t^{2}+\frac{2\pi nm}{N}+Cm^{2}\Delta \omega^{2}+Dn\Delta t+Em\Delta \omega \right)\right\}\\ \; & \;=\frac{1}{\sqrt{N-U}}\sum_{n=0}^{N-1}\;\sum_{v=0}^{N-U-1}x\left(u\right)\;y\left(v\right)\exp\left\{iAv\left(v+2u\right)\;\Delta t^{2}\right\}\\ \; & \;\;\times \exp\left\{-i\left(A{\left(u+v\right)}^{2}\Delta t^{2}+\frac{2\pi (u+v)m}{N}+Cm^{2}\Delta \omega^{2}+D\left(u+v\right)\Delta t+Em\Delta \omega \right)\right\}\\ \; & \;=\sum_{n=0}^{N-1}x\left(u\right)\exp\left\{-i\left(Au^{2}\Delta t^{2}+\frac{2\pi um}{N}+Cm^{2}\Delta \omega^{2}+Du\Delta t+Em\Delta \omega \right)\right\}\\ \; & \;\;\times \frac{1}{\sqrt{N-U}}\sum_{u=0}^{N-U-1}e^{-iDv\Delta t}\;y\left(v\right)\exp\left\{\frac{-i2\pi vm}{N}\right\}\\ \; & \;=\sqrt{N}\;L_{\Lambda }\left(x_{N}\right)\left(m\right)\;F\left(e^{-iDn\Delta t}\;y_{N}\right)\left(m\right), \end{array}$$

where $F\left(x_{N}\right)\left(m\right)$ denotes the classical discrete Fourier transform.

This completes the proof of Theorem 8.  □

**Remark** **1.** *For $\Lambda =\left(0,1,0,0,0\right)$, Definition 5 yields the classical convolution operator and the corresponding convolution theorem is obtained from* (17)*.*

Towards the culmination, we introduce the notion of chirp-free discrete quadratic-phase correlation and then present the corresponding correlation theorem.

**Definition** **6.** Given a pair of sequneces $x,y\in \ell^{2}\left(Z_{N}\right)$, the chirp-free discrete quadratic-phase correlation ${*}_{\Lambda }$ is defined as

(18) $$\begin{array}{c} \left(x_{N}{*}_{\Lambda }y_{N}\right)\left(u\right)=\sum_{n=0}^{N-1}x\left(n\right)\;\bar{y(n-u)}\;\exp\left\{iA\left(u^{2}-n^{2}\right)\Delta t^{2}\right\}. \end{array}$$

**Theorem** **9.** *For any $x,y\in \ell^{2}\left(Z_{N}\right)$, we have*

(19) $$\begin{array}{c} L_{\Lambda }\left(x_{N}{*}_{\Lambda }y_{N}\right)\left(m\right)=\sqrt{N}\;L_{\Lambda }\left(x_{N}\right)\left(m\right)\;\bar{F\left(e^{-iDn\Delta t}\;y_{N}\right)\left(m\right)}. \end{array}$$

**Proof.**  The proof can be obtained in a manner similar to Theorem 5 and is therefore omitted.  □

## 5. Conclusions

In this article, we have introduced the notion of discrete quadratic-phase Fourier transform and studied its fundamental properties. In continuation, we formulated a weighted-type convolution and correlation structures associated with the discrete QPFT. Next, we established a chirp-free discrete convolution and product theorems in the QPFT domain. Finally, we showed that such convolution is non-commutative and non-associative but inhibits a distributive property. 

## Data Availability

The data used to support the findings of this study are included within the article.
